# Associations between data-driven lifestyle profiles and cognitive function in the AusDiab study

**DOI:** 10.1186/s12889-022-14379-z

**Published:** 2022-11-29

**Authors:** Sara E Dingle, Steven J Bowe, Melissa Bujtor, Catherine M Milte, Robin M Daly, Kaarin J Anstey, Jonathan E Shaw, Susan J Torres

**Affiliations:** 1 Institute for Physical Activity and Nutrition, School of Exercise and Nutrition Sciences, Deakin University, Geelong, VIC Australia; 2 Biostatistics Unit, Faculty of Health, Deakin University, Geelong, VIC Australia; 3 Stress, Psychiatry and Immunology Laboratory, Department of Psychological Medicine, Institute of Psychiatry, Psychology & Neuroscience, King’s College, SE5 9RT London, UK; 4 University of New South Wales, Sydney, NSW Australia; 5 Neuroscience Research Australia, Sydney, NSW Australia; 6 Baker Heart and Diabetes Institute, PO Box 6492, 3004 Melbourne, VIC Australia

**Keywords:** Australian adults, Lifestyle patterns, Cognition, Latent Profile Analysis, Data-driven

## Abstract

**Background:**

Mounting evidence highlights the importance of combined modifiable lifestyle factors in reducing risk of cognitive decline and dementia. Several *a priori* additive scoring approaches have been established; however, limited research has employed advanced data-driven approaches to explore this association. This study aimed to examine the association between data-driven lifestyle profiles and cognitive function in community-dwelling Australian adults.

**Methods:**

A cross-sectional study of 4561 Australian adults (55.3% female, mean age 60.9 ± 11.3 years) was conducted. Questionnaires were used to collect self-reported data on diet, physical activity, sedentary time, smoking status, and alcohol consumption. Cognitive testing was undertaken to assess memory, processing speed, and vocabulary and verbal knowledge. Latent Profile Analysis (LPA) was conducted to identify subgroups characterised by similar patterns of lifestyle behaviours. The resultant subgroups, or profiles, were then used to further explore associations with cognitive function using linear regression models and an automatic Bolck, Croon & Hagenaars (BCH) approach.

**Results:**

Three profiles were identified: (1) “Inactive, poor diet” (76.3%); (2) “Moderate activity, non-smokers” (18.7%); and (3) “Highly active, unhealthy drinkers” (5.0%). Profile 2 “Moderate activity, non-smokers” exhibited better processing speed than Profile 1 “Inactive, poor diet”. There was also some evidence to suggest Profile 3 “Highly active, unhealthy drinkers” exhibited poorer vocabulary and verbal knowledge compared to Profile 1 and poorer processing speed and memory scores compared to Profile 2.

**Conclusion:**

In this population of community-dwelling Australian adults, a sub-group characterised by moderate activity levels and higher rates of non-smoking had better cognitive function compared to two other identified sub-groups. This study demonstrates how LPA can be used to highlight sub-groups of a population that may be at increased risk of dementia and benefit most from lifestyle-based multidomain intervention strategies.

**Supplementary information:**

The online version contains supplementary material available at 10.1186/s12889-022-14379-z.

## Background

Dementia is a key concern for our ageing population, estimated to affect approximately 50 million people world-wide, with almost 10 million new cases per year [1]. In Australia, dementia is the second leading cause of death [2] and is associated with a significant financial burden for governments, communities, families and individuals [3]. Due to the progressive and currently incurable nature of dementia, greater focus needs to be directed at factors that may slow preclinical cognitive decline to ultimately reduce incident dementia at the population level. Assessing pre-clinical cognitive performance can be employed as a marker of risk for neurocognitive disorders such as mild cognitive impairment (MCI) and dementia, with domains such as memory, processing speed and language forming a subset of the diagnostic factors for such conditions [4].

Non-modifiable risk factors such as age and genetics are strong determinants of neurocognitive disorders [5]. However, there is increasing evidence that modifiable factors can impact cognitive outcomes [1], with such factors estimated to account for up to 40% of dementia risk [6]. Poor nutrition, excess alcohol consumption, smoking and physical inactivity have been identified as key health-related behaviours (HRBs) impacting on cognitive function and risk of late-life dementia [7–13]. These HRBs, collectively known as ‘SNAP’ risk factors [14, 15], are not only key in the context of neurodegeneration but have been identified as leading causes of morbidity and mortality worldwide [16, 17]. Various nutrients, food items and dietary patterns have been linked to a reduced risk of neurocognitive disorders [7, 8]. The MIND (Mediterranean-DASH Intervention for Neurodegenerative Delay) diet, characterised by increased consumption of brain-healthy foods such as berries and leafy greens, and lower intakes of foods such as red meat and discretionary items has been linked to better cognitive function and reduced risk for cognitive impairment and dementia [18–21]. These effects are likely due to the impact of dietary factors on inflammation, oxidative stress and vascular health, all risk factors for cognitive decline [22]. There is also strong evidence to support the benefits of regular physical activity (PA) for brain health in mid- and late-life [9]. Possible mechanisms attributed to this effect include the impact of PA on lowering psychological stress, ameliorating vascular and metabolic risk factors, and increasing cognitive reserve, brain volume, brain-derived neurotrophic factor (BDNF) and amyloid clearance [22]. Smoking is an established risk factor for cognitive decline and dementia [13, 23], likely because of resultant increases in oxidative stress and other effects on inflammatory, vascular and degenerative processes [24]. The alcohol-cognition link is nuanced with excessive alcohol consumption linked to increased risk for neurodegenerative conditions, yet some evidence indicates that light-moderate alcohol consumption may be protective against dementia [11, 12]. Excess alcohol consumption likely has implications for dementia risk through several mechanisms including neuroinflammation, neurotoxicity, nutritional deficiency, amyloid aggregation and changes in neurotransmitter systems [25]. Possible protective effects may be attributed to intakes of specific forms of alcohol, such as wine, and the presence of flavonoids [22].

Much research has examined the role of individual HRBs in association with cognition and neurodegenerative conditions. However, it is more likely that these behaviours co-occur and/or cluster [26–28]. Understanding the clustering of key HRBs and associations with brain health may provide important insights to inform population interventions to improve HRBs and reduce risk of neurodegenerative conditions such as dementia. An increasing number of studies have explored the association between combined modifiable risk factors and cognition [29], however, majority have employed additive index-based approaches, whereby individual risk factors are summed to provide a total score. This results in a score that is simple to interpret and aggregate. However, many of the commonly included lifestyle factors are interrelated and thus may be more likely to act in a multiplicative or synergistic manner, rather than following an additive relationship. An index-based approach facilitates exploration of the dose-response association between modifiable risk factors and cognitive outcomes, but it is generally not possible to discern between specific combinations of factors. Data-driven person-centred approaches such as Latent Class Analysis (LCA) and Latent Profile Analysis (LPA) account for underlying associations between HRBs and enable detailed characterisation of identified sub-groups.

Whilst it is the case that unhealthy behaviours such as smoking and alcohol consumption often cluster together in adult populations [15, 28, 30], there is also evidence to suggest clustering of heterogenous combinations of HRBs. For instance, a previous study in a Dutch population found significant clustering between all combinations of smoking, fruit and vegetable intake, excessive alcohol, and low PA, with the exception for low PA and excessive alcohol consumption [30]. Similarly, in an English population, it was reported that the combination of heavy drinking and lack of activity had a lower than expected prevalence [28]. As previous literature has indicated, healthy HRBs do not always cluster with other healthy HRBs and unhealthy HRBs do not always cluster with other unhealthy HRBs. Heterogenous groups are commonly observed. This is where advanced data-driven approaches can assist and go beyond the dose-response type findings that can be observed through an additive index-based approach.

Employing LPA to provide person-centred insights into clustering of key HRBs (diet, PA, smoking, alcohol) and associations with cognition in a general Australian population may provide important insights to assist in policy development, identification of at-risk subgroups, and refinement of existing strategies and recommendations. To the best of the authors knowledge, no studies to date have explored clustering of key HRBs in a large representative Australian sample and associations with cognitive function. Therefore, the aims of this study were to identify sub-groups, or profiles, of individuals based on lifestyle-related behaviours and to examine associations between these profiles and cognitive function in community-dwelling Australian adults.

## Methods

### Study population

The Australian Diabetes, Obesity and Lifestyle Study (AusDiab) is a large national population-based study with the primary purpose of determining the prevalence and incidence of obesity, diabetes and other cardiovascular disease risk factors in Australian adults aged ≥ 25 years [31]. A detailed description of the methodology has been reported elsewhere [31]. Briefly, baseline data were collected in 1999–2000 and included 11,247 Australian adults (5,049 men, 6,198 women). Sample selection involved stratified cluster sampling from the six states and the Northern Territory based on census collector districts. For all participants, information was collected on demographics, medical and family history, physical measurements, and lifestyle behaviours, with blood and urine measurements also included. A follow-up was conducted in 2011-12 in 4,614 participants (2,062 men; 2,552 women). Cognitive assessment was conducted for consenting participants (n = 4,561) who attended survey sites in the third wave of data collection (2011-12). Written informed consent was obtained from all participants prior to commencing the study. Ethics approval was obtained from the ethics committees of the International Diabetes Institute, Monash University and Alfred Hospital [32]. An ethics exemption was also granted by the Deakin University Human Research Ethics Committee (DUHREC) for the use of the data in this manuscript (2022-089). All participants were de-identified. The current study involves a secondary cross-sectional analysis of information obtained at the 2011-12 follow-up to assess the association between lifestyle profiles and cognitive function.

### Lifestyle behaviours

#### Diet

A validated Food Frequency Questionnaire (FFQ) developed by the Anti-Cancer Council of Victoria (CCV) was used to collect data on dietary intake [33]. For each item on the FFQ, there were 10 possible frequency options ranging from *‘never’* to *‘3 or more times per day’*. MIND diet scores were calculated. The MIND diet combines the Mediterranean and DASH diet with a particular focus on dietary components that are reported to be neuroprotective [20]. The MIND diet is comprised of 15 components: 10 brain healthy foods (green leafy vegetables, other vegetables, nuts, berries, whole-grains, fish, poultry, olive oil and wine) and five less healthy foods (red meat, butter/margarine, cheese, pastries and sweets, and fried/fast food). Each MIND diet component was scored a 0, 0.5 or 1 according to the methodologies employed in previous studies [20, 21] and a total MIND diet score was calculated by adding the individual component scores. The CCV FFQ did not include questions for butter/margarine or olive oil consumption as separate items, so these were omitted from the MIND score calculation in the current study. The possible range of MIND scores in the current sample was 0–13, which aligns with an established approach [19]. Increasing scores reflect better adherence and healthier diet.

#### Physical activity

A validated self-reported questionnaire, the Active Australia Survey (AAS), was used to determine PA in the previous 7 days [34]. The survey includes eight questions to assess participation in a variety of activities (e.g., walking, vigorous gardening or heavy work around the yard, vigorous PA). The AAS has been shown to provide a reliable and valid estimate of PA among adults [35]. As previously reported [36, 37], total PA was calculated by summing the time spent walking (if continuous and > 10 min), the time spent doing moderate-intensity activities plus the time spent participating in vigorous PA (doubled). Total PA (hours per week) > 1680 min (28 h) were recoded to 1680 min (28 h) to avoid errors due to over-reporting, in line with the Active Australia Manual [34].

#### Sedentary status

Sedentary status was assessed by total sitting time with participants self-reporting sitting time, separately for weekdays and weekend days, over the previous 7 days, across five different contexts (occupational, transport, television viewing, leisure-time computer use and “other” sitting). These questions were devised for the AusDiab study specifically and the total sitting time (sum of each of the five contexts) has previously been validated against total sitting time measured by activPAL (*r* = 0.46 [95% CI: 0.40, 0.52]) [38]. The average total screen time for a weekday and a weekend were calculated and those with implausible sitting time on a weekday or weekend day (> 18 h) were recoded to 18 h to avoid errors due to over-reporting [39]. A total weighted average daily sitting time (h/day) was then calculated as the sum of all forms of sitting and then applying the following equation [(hours/day average weekday*5 + hours/day average weekend*2)/7)].

#### Smoking

Smoking status was divided into three categories: “current smoker”, “former smoker” or “non-smoker”.

#### Alcohol

Alcohol consumption was assessed using responses to the CCV FFQ. Participants were asked ‘Over the last 12 months, how often did you drink beer, wine and/or spirits?’ with response options ranging from *‘never’* to *‘every day’*. Participants were also asked ‘Over the last 12 months, on days when you were drinking, how many glasses of beer, wine and/or spirits altogether did you drink?’ with response options ranging from 1 to ≥10. The participants were prompted to convert the amount they usually consumed into standard drinks with the help of conversion examples. These data were converted into a continuous measure expressed in grams of ethanol per day [40]. Participants were also asked to indicate the maximum number of drinks they consume in a 24-hour period with the following question “Over the last 12 months, what was the *maximum* number of glasses of beer, wine and/or spirits that you drank in 24 hours?”. Alcohol consumption was then divided into three categories based on the most recent National Health and Medical Research Council (NHMRC) recommendations for alcohol consumption in adults [41]: “Non-drinkers” (no alcohol consumed), “Drinkers meeting recommendations” (adhering to the recommendation of no more than 10 standard drinks (100 g ethanol/week) and no more than 4 standard drinks on any one day) and “Drinkers above recommendations” (exceeding the recommendations, i.e., > 10 drinks per week and/or more than 4 drinks in a 24-hour period). This categorisation is in line with the World Health Organisation (WHO) Guidelines for Risk Reduction of Cognitive Decline and Dementia, citing extensive evidence on excessive alcohol as a risk factor for dementia and cognitive decline [1].

### Cognitive function

Vocabulary and verbal knowledge was tested using the Spot-the-Word (STW) test [42]. This test involved presenting participants with pairs of words and instructing them to identify the real word when presented with a real word and a false word. If correctly identified a score of one was allocated. Participants were presented with 60 pairs resulting in a total score ranging from 0 to 60. The California Verbal Learning Test (CVLT) was used to assess memory [43]. This test involved participants listening to a list of 16 common shopping list items read by the interviewer and subsequently repeating as many of these items as possible (immediate recall). After 20 min, participants were again asked to recall as many items as they could (delayed recall). For each item correctly recalled a score of one was allocated, resulting in a total score ranging from 0 to 16. Processing speed was assessed using the Symbol-Digit Modalities Test (SDMT) [44]. Participants were provided with a reference key and allowed 90 s to match geometric figures with specific numbers. The total score ranged from 0 to 120.

### Confounders and other variables of interest

Age, sex, education, and country of birth were self-reported. Participants were asked to specify their highest levels of education completed and responses were coded into three categories: (a) none, primary or some high school, (b) completed high school, year 12 or equivalent, or (c) completed university, TAFE, or equivalent. Previous classifications for ethnicity utilised in this dataset, characterised by ‘Europid’ or ‘non-Europid’, were employed [45, 46]. Europids included those both in Australia, Canada, USA, New Zealand and Northern Europe, whereas non-Europids included individuals born in Southern Europe, the Middle East, Asia, India and Sri Lanka, Africa, Pacific Islands, and South and Central America. Aboriginal Australians and Torres Strait Islanders were also classified as non-Europids. Data on country of birth data was obtained from baseline (1999–2000).

Depression was assessed using a shortened version of the Centre for Epidemiologic Studies Depression Scale (CES-D), comprising 10 questions focused on recent feelings and behaviours over the previous week [47]. Height and weight were measured using standard techniques from which BMI was calculated (weight (kg) divided by height (m)^2^). Participants were classified as having diabetes if they reported having doctor-diagnosed diabetes and were either taking hypoglycaemic medication or had a fasting plasma glucose (FPG) ≥7 mmol/l or 2-hour PG ≥11.1 mmol/l. Blood pressure (BP) measurements were performed while seated (after 5 min rest) using an automated monitor (Dinamap Pro-Series Monitor Model DP 101-NIBP, pulse and recorder; GE Systems, Friburg, Germany). The average of two BP measurements was calculated. Hypertension was coded as a binary variable and defined as having a systolic BP (SBP) of ≥ 140 mmHg and/or ≥ 90 diastolic BP (DBP) mmHg or those on anti-hypertensive medication [48]. Presence of cardiovascular disease (CVD) was identified as anyone who self-reported being told by a doctor or nurse that they have had angina and/or heart attack.

### Statistical analyses

#### Latent profile analysis

Latent profile analysis (LPA) was performed in *Mplus* Version 8.6 (Muthen & Muthen, Los Angeles, CA, USA) with full information maximum likelihood estimation to identify distinct lifestyle profiles [49]. Models with 1–6 profiles were tested, and fit indices examined to determine most appropriate profile solution including: Akaike Information Criterion (AIC), Bayesian Information Criterion (BIC) and adjusted BIC (lower values indicating better model fit); entropy (range 0–1, with values closer to 1 indicating better class separation); Lo-Mendell-Rubin Likelihood Ratio test (LMR-LRT) and bootstrapped likelihood ratio test (BLRT) value [50]. The LMR-LRT and BLRT tests compare the fit of a target model (k-profiles) to a model that specifies one fewer class (k-1 profiles). The “higher class” (k) solution is considered superior when p values < 0.05, whereas the “lower class” (k-1) solution is considered superior when p > 0.05. Along with consideration of these fit indices the final decision on number of profiles selected was informed by interpretability, preferring a model whose profile separation was the simplest to articulate, and for which relative size of profiles was no lower than approximately 5% of the sample [50].

#### Associations with cognitive function

After determining the optimal number of profiles, we exported the profile assignment based on highest probability classification into Stata/SE version 17 [51]. For further characterisation of profiles, associations between profile membership and several key variables were examined using chi-square tests for categorical variables and one-way ANOVAs with multiple pairwise comparisons using a Bonferroni adjustment for continuous variables. Associations between profile membership and cognitive function were examined using linear regression models with case-wise exclusion for missing data. Cognitive test scores were standardised prior to inclusion in both unadjusted and adjusted models to allow for cross-comparison across tests. Confounders/covariates included in the model were determined by the use of a directed acyclic graph (DAG) (DAGitty version 2.3) (refer to Supplementary Fig. 1 ), informed by background literature [52]. Models were adjusted for age, sex, education and ethnicity. Other risk factors such as BMI, hypertension, depression, CVD, diabetes that likely lie on the causal pathway were omitted from the adjusted model. These are considered intermediary variables in our DAG, and overadjustment for such variables can be considered to reflect overadjustment bias [53]. As part of sensitivity analyses we also employed the automatic BCH approach to explore pairwise comparisons between cognitive test scores across classes [54]. The BCH approach accounts for possible class/profile classification error by using a weighted multiple group analysis to evaluate means across classes/profiles for a continuous auxiliary variable. The BCH method has been shown to outperform other methods and avoid shifts in latent classes with the introduction of auxiliary variables [54].

## Results

The analytic sample comprised 4561 participants (55.3% female, mean age 60.9 ± 11.3 years) with cognitive data at the third wave of the AusDiab follow-up. Compared to those missing from baseline (1999–2000, N = 6,686), the analytic sample did not differ in regard to gender distribution, however they were on average, younger (mean 48.9 vs. 53.3 years, p < 0.0001); less likely to be current daily smokers (10.1% vs. 17.5%, p < 0.001); more likely to be physically active (4.7 vs. 4.4 h/week, p < 0.01), have a higher consumption of alcohol (13.5 vs. 11.7 g/day, p < 0.0001); have better adherence to the MIND diet (6.5 vs. 6.3, p < 0.0001); exhibit lower body mass index (BMI) (26.6 vs. 27.3, p < 0.0001); less likely to have CVD (4.1% vs. 11.4%, p < 0.001) and less likely to have diabetes (5.5% vs. 12.0%, p < 0.001).

### Latent profile analysis

Model fit statistics for each model are shown in Table 1. The information criteria (i.e., AIC, BIC, aBIC) decreased when number of latent profiles was increased. Log-likelihood also increased according to increased number of profiles. Entropy was close to 0.9 for 2–5 class approaches, indicating that individuals are well-classified into profiles [50]. Profile proportions were also considered whereby all groups should constitute at least 5% of the total sample [50]. Based on consideration of model fit indices, interpretability of profiles and profile proportions, the three-profile solution was selected for all subsequent analyses.

**Table 1:** Model fit statistics for each of the fitted Latent Profile Analysis (LPA) models

Statistic	1 profile	2 profiles	3 profiles	4 profiles	5 profiles	6 profiles
Log-likelihood	-40613.06	-39871.65	-39498.89	-39316.81	-39158.07	-39082.47
FP	10	18	26	34	42	50
AIC	81246.11	79894.90	79049.78	78701.61	78400.14	78264.93
BIC	81310.34	79894.90	79216.76	78919.98	78669.88	78586.05
adjusted BIC	81278.56	79837.70	79134.14	78811.94	78536.42	78427.17
Entropy	N/A	0.90	0.88	0.86	0.88	0.77
^a^LMR-LRT	N/A	1461.13, *P* < 0.0001	734.62, *P* < 0.0001	358.84, *P* < 0.0001	312.83, *P* < 0.0001	149.00, *P* = 0.139
BLRT	N/A	*P* < 0.0001	*P* < 0.0001	*P* < 0.0001	*P* < 0.0001	*P* < 0.0001
Class proportions	100%	88.5%, 11.5%	76.3%, 18.7%, 5%	62.6%, 23.9%, 9.5%, 4.0%	59.1%, 24.1%, 10.4%, 3.8%, 2.6%	33.3%, 25.0%, 25.0%, 10.4%, 3.8%, 2.6%

*AIC* Akaike Information Criterion, *BIC* Bayesian Information Criterion, *BLRT* Bootstrapped-Likelihood Ratio Test, *FP* Free Parameters, *LMR* Lo-Mendell-Rubin, *LRT* likelihood ratio test

^a^Adjusted Lo-Mendell-Rubin likelihood ratio test for k versus k-1 profiles. Values are two times the loglikelihood difference and corresponding *p*-value

### Lifestyle behaviours across profiles

The estimates for the lifestyle behaviours (means for continuous variables and response probabilities for categorical variable) within each of the three classes are presented in Table 2. Profile 1 was the most common group, comprising 76.3% of the sample. This group was labelled “Inactive, poor diet”, demonstrating the lowest MIND diet score, lowest time spent doing PA, highest sedentary time, highest probabilities of being a non-drinker or drinker meeting recommendations (14.2% and 50.3%, respectively) and highest probability of being a current smoker (5.9%) (Table 2). Profile 2 comprised 18.7% of the sample and was labelled “Moderate activity, non-smokers”. This group had the highest MIND diet score (although very similar to Profile 3), moderate PA levels, moderate sedentary time, moderate probability of being a drinker meeting recommendations (48.6%) and highest probability of being a ‘never smoker’ (60.7%). Profile 3 was the smallest group, comprising only 5.0% of the sample and was labelled “Highly active, unhealthy drinkers” (Table 2). This group had a moderate MIND diet score (although very similar to Profile 2), high levels of PA, lowest sedentary time, highest probability of being a drinker exceeding recommendations (46.5%) and lowest probability of being a ‘never smoker’ (55.5%).

Additional analyses were carried out to explore the contribution of each activity component (walking, moderate PA, vigorous PA) to total mean PA values. In relation to vigorous PA, Profile 3 exhibited approximately 6.6 h per week compared to < 0.5 h per week for Profile 1 and approximately 2.3 h per week for Profile 2. In terms of moderate PA, Profile 3 engaged in approximately 4.2 h per week compared to < 0.5 and approximately 2.3 h per week for Profile 1 and Profile 2, respectively. Lastly, for walking time, the mean for Profile 1 was just under 2 h per week, approximately 5.5 h per week for Profile 2 and around 8 h per week for Profile 3.

**Table 2:** Estimated response means (for continuous variables) and probabilities (for categorical variables) in each lifestyle profile

Lifestyle behaviour	Profile 1 “Inactive, poor diet” n = 3472 (76.3%)	Profile 2 “Moderate activity, non-smokers” n = 850 (18.7%)	Profile 3 “Highly active, unhealthy drinkers” n = 226 (5.0%)
MIND^a^	6.82 [6.77, 6.87]	7.19 [7.07, 7.30]	7.17 [6.97, 7.37]
PA (hours/week)	3.03 [2.88, 3.18]	11.91 [11.45, 12.36]	23.77 [23.04, 24.50]
Sedentary time (hours/day)	6.85 [6.75, 6.94]	6.49 [6.29, 6.69]	6.33 [5.97, 6.68]
Non-drinker	0.14 [0.13, 0.15]	0.09 [0.07, 0.12]	0.10 [0.06, 0.14]
Drinker meeting recommendations	0.50 [0.49, 0.52]	0.49 [0.45, 0.53]	0.43 [0.37, 0.50]
Drinker exceeding recommendations	0.36 [0.34, 0.37]	0.42 [0.38, 0.46]	0.47 [0.39, 0.53]
Current smoker	0.06 [0.05, 0.06]	0.05 [0.03, 0.06]	0.05 [0.02, 0.08]
Ex-smoker	0.35 [0.33, 0.37]	0.34 [0.31, 0.38]	0.39 [0.33, 0.46]
Never smoker	0.59 [0.53, 0.61]	0.61 [0.57, 0.64]	0.56 [0.49, 0.62]

^a^MIND scores range from 0–13, with higher scores indicating greater adherence

Figures presented as mean [95% CI] for continuous variables and probability [95% CI] for categorical variables

### Sample characteristics across lifestyle profiles

Profile 3 had a significantly lower proportion of females compared to Profiles 1 and 2 (Table 3). There were no significant differences in mean age across profiles. Profile 3 had the lowest proportion of non-Europids (1.8%), followed by Profile 2, with Profile 1 exhibiting the highest proportion (9.0%). Profile 3 had the highest proportion of individuals who had completed the highest level of education, i.e., university, TAFE or equivalent and highest income bracket ($80,000+), followed by Profile 2 and then Profile 1. BMI was highest in Profile 1 at 28.1 kg/m^2^, followed by Profile 2, with Profile 3 exhibiting the lowest mean BMI at 25.8 kg/m^2^. Profile 2 had the lowest proportion of individuals with hypertension, CVD, and diabetes. In terms of depression scores (CESD) both Profile 2 and Profile 3 had significantly lower mean scores compared to Profile 1.

**Table 3:** Sample characteristics within each lifestyle profile (using final 3-profile model approach)^a^

	Profile 1 “Inactive, poor diet” n = 3472 (76.3%)	Profile 2 “Moderate activity, non-smokers” n = 850 (18.7%)	Profile 3 “Highly active, unhealthy drinkers” n = 226 (5.0%)	Comparison	p-value
**Female, n (%)**	2009 (57.9%)	413 (48.6%) ^^^	93 (41.2%) ^^*^	χ^2^ (2): 43.02	< 0.001
**Age in years, mean (SD)**	60.9 (11.5)	60.2 (10.5)	59.7 (10.1)	F (2, 4545): 2.48	0.084
**Highest level of education, n (%)**				χ^2^ (4): 18.91	0.001
None, primary or some high school	987 (33.1%)	194 (27.0%) ^^^	47 (23.6%) ^^^		
Completed high school, year 12 or equivalent	676 (22.7%)	166 (23.1%) ^^^	43 (21.6%) ^^^		
Completed University, TAFE or equivalent	1317 (44.2%)	358 (49.9%) ^^^	109 (54.8%) ^^^		
**Income, n (%)**				χ^2^ (4): 20.58	< 0.001
<$49,999	938 (30.5%)	195 (24.6%) ^^^	41 (19.6%) ^^^		
$49,999 - $79,999	855 (27.8%)	236 (29.8%) ^^^	61 (29.2%) ^^^		
$80,000+	1284 (41.7%)	361 (45.6%) ^^^	107 (51.2%) ^^^		
**Ethnicity**				χ^2^ (2): 21.24	< 0.001
Europid	3152 (91.0%)	799 (94.0%) ^^^	222 (98.2%) ^^*^		
Non-Europid	313 (9.0%)	51 (6.0%) ^^^	4 (1.8%) ^^*^		
**BMI (kg/m** ^**2**^ **), mean (SD)**	28.1 (5.4)	26.9 (4.6) ^^^	25.8 (4.1%) ^^*^	F (2, 4540): 32.25	< 0.0001
**Hypertension, n (%)**	1531 (44.2%)	299 (35.3%) ^^^	86 (38.1%)	χ^2^ (2): 23.87	< 0.001
**CVD, n (%)**	150 (4.6%)	29 (3.5%)	8 (3.7%)	χ^2^ (2): 1.84	0.399
**Diabetes, n (%)**	147 (4.2%)	23 (2.7%) ^^^	6 (2.7%)	χ^2^ (2): 5.24	0.073
**CESD depression score, mean (SD)** ^**b**^	4.3 (4.6)	3.6 (4.0) ^^^	3.0 (3.8) ^^^	F (2, 4434): 16.74	< 0.0001

^a^Results are presented based on highest probability profile assignment

^b^CESD scores ranged from 0–30 with higher scores indicating increased risk of depression

Post hoc testing: ^ Significant difference from Profile 1 (p < 0.05), * Significant difference from Profile 2 (p < 0.05)

### Associations between lifestyle profiles and cognitive function

Results from linear regression models between profile assignment (based on highest probability classification) and cognitive test scores (standardised) are presented in Table 4. Adjusted models included key confounders age, sex, ethnicity, and education. Profile 2 *‘Moderate activity, non-smokers’* exhibited significantly better processing speed than Profile 1 *‘Inactive, poor diet’* in unadjusted models (p = 0.008) but fell just short in adjusted (p = 0.050). Profile 3 *‘Highly active, unhealthy drinkers’* exhibited significantly poorer vocabulary and verbal knowledge compared to Profile 1 in adjusted models (p = 0.029). Profile 3 also demonstrated significantly poorer processing speed and memory scores compared to Profile 2 in adjusted models (p = 0.018 and p = 0.024, respectively).

**Fig. 1:**
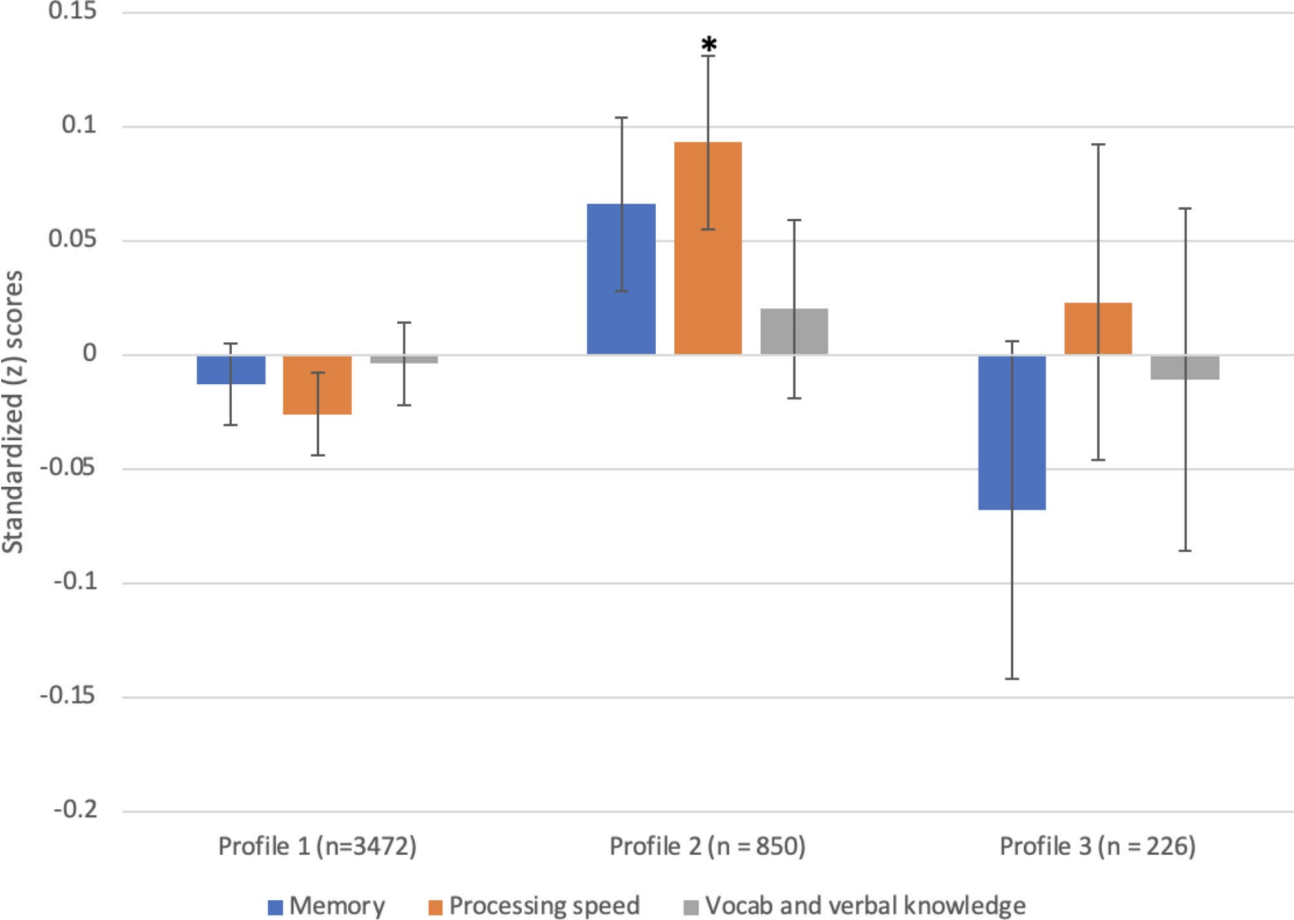
Standardised cognitive test scores across three lifestyle profiles using the automatic BCH procedure Error bars represent standard error (SE) *Significant difference from Profile 1 (p < 0.05) *BCH*: Bolck, Croon, and Hagenaars

An automatic BCH approach was also employed to test for mean differences across class cognitive test scores, accounting for possible classification error [54]. Standardised mean memory score was − 0.0087 for Profile 1, 0.0541 for Profile 2 and − 0.0688 for Profile 3 (Fig. 1). Standardised mean processing speed scores were − 0.0213 for Profile 1, 0.0808 for Profile 2, and 0.0201 for Profile 3. Standardised mean scores score for vocabulary and verbal knowledge were − 0.0051 for Profile 1, 0.0227 for Profile 2 and − 0.0089 for Profile 3. Pairwise comparisons demonstrated that Profile 2 *‘Moderate activity, non-smokers’* exhibited significantly better processing speed than Profile 1 *‘Inactive, poor diet’* but there were no significant differences between profiles 1 and 3 or between profiles 2 and 3. In terms of both vocabulary and verbal knowledge and memory, no significant differences across profiles were observed.

**Table 4:** Associations between lifestyle profiles and cognitive function (using standardised cognitive test scores, z-scores)

		Memory (CVLT), n = 4502		Processing speed (SDMT), n = 4511		Vocab & Verbal knowledge (STW), n = 4502	
		**Unadjusted** **β [95% CI]**	**Adjusted** **β [95% CI]**	**Unadjusted** **β [95% CI]**	**Adjusted** **β [95% CI]**	**Unadjusted** **β [95% CI]**	**Adjusted** **β [95% CI]**
*Profile 1 (Reference Group)*	Profile 2	0.06 [-0.01, 0.14]	0.06 [-0.01 0.14]	0.10 [0.03, 0.18] **	0.06 [0.00, 0.12]	0.03 [-0.05, 0.10]	-0.00 [-0.08, 0.07]
	Profile 3	-0.06 [-0.19, 0.07]	-0.10 [-0.23, 0.03]	0.04 [-0.09, 0.18]	-0.08 [-0.19, 0.03]	-0.00 [-0.14, 0.13]	-0.15 [-0.29, -0.01] *
*Profile 2 (Reference Group)*	Profile 1	-0.06 [-0.14, 0.01]	-0.06 [-0.14, -0.01]	-0.10 [-0.18, -0.03] **	-0.06 [-0.12, -0.00]	-0.03 [-0.10, 0.05]	0.01 [-0.07, 0.08]
	Profile 3	-0.12 [-0.27, 0.02]	-0.16 [-0.30, -0.02] *	-0.06 [-0.21, 0.09]	-0.14 [-0.26, -0.02] *	-0.03 [-0.18, 0.12]	-0.14 [-0.29, 0.00]
*Profile 3 (Reference Group)*	Profile 1	0.06 [-0.07, 0.19]	0.10 [-0.03, 0.23]	-0.04 [-0.18, 0.09]	0.08 [-0.03, 0.19]	0.00 [-0.13, 0.14]	0.15 [0.01, 0.29] *
	Profile 2	0.12 [-0.02, 0.27]	0.16 [0.02, 0.30] *	0.06 [-0.09, 0.21]	0.14 [0.02, 0.26] *	0.03 [-0.12, 0.18]	0.14 [-0.00, 0.29]

Profile 1 “Inactive, poor diet” (n = 3472), Profile 2 “Moderate activity, non-smokers” (n = 850), Profile 3 “Highly active, unhealthy drinkers” (n = 226)

Adjusted models include age, sex, education and ethnicity

β: standardised regression coefficient, CI: Confidence Interval, CVLT: California Verbal Learning Test, SDMT: Symbol-Digit Modalities Test, STW: Spot-the-word test

*p < 0.05; **p < 0.01

## Discussion

This study aimed to identify similar sub-groups, or profiles, of individuals based on five lifestyle-related behaviours and to examine associations between these profiles and cognitive function in community-dwelling Australian adults. Three distinct profiles were identified in this study: “Inactive, poor diet” (Profile 1, 76.3%), “Moderate activity, non-smokers” (Profile 2, 18.7%), and “Highly active, unhealthy drinkers” (Profile 3, 5.0%). With respect to the small sub-group characterised primarily by very high PA levels and the highest probability of being classified as exceeding alcohol consumption recommendations, similar findings have been shown in a representative sample of Japanese adults [55]. This study identified a small cluster characterised by higher PA levels (measured by steps per day) and a high proportion of participants exceeding alcohol guidelines in both men and women [55]. Similar findings can also be drawn from an English population, whereby lack of PA was inversely clustered with both smoking and heavy drinking [28]. A potential explanation for this finding may be related to type of occupation, whereby individuals with a manual occupation are more likely to drink heavily but also engage in high levels of habitual PA [28].

Use of continuous input variables where possible (MIND score, PA, sedentary status) enabled novel insights into the kinds of behavioural profiles observed in sub-groups of this Australian community-dwelling sample. For example, we were able to distinguish between a group (Profile 2, 18.5% of total sample) that engages in a higher weekly amount of PA (approximately 12 h per week) versus a small subgroup (Profile 3, 5% of total sample) that is extremely active (mean approximately 24 h of PA per week). Whilst both these levels of weekly PA appear very high, it should be acknowledged that vigorous PA has been doubled (as per the survey guidelines to reflect the additional health benefits associated with more intense activity) and that this total is also inclusive of weekly walking time. Further exploratory analyses to explore the contribution of each activity type to total PA revealed that vigorous PA was considerably higher in Profile 3 (approximately 6.6 h per week) compared to Profiles 1 (< 0.5 h per week) and Profile 2 (approximately 2.3 h per week). On the other side of the spectrum, the use of LPA in this study highlights that in this population of Australian adults there is a large subgroup (76.3%, Profile 1) exhibiting low levels of PA and as such represents an opportunity for improvement and perhaps a group to which intervention efforts should be prioritised. The mean PA for this subgroup was 3 h per week but when examining the contribution of each activity type, it was observed that mean levels for moderate PA and vigorous PA were each less than 30 min per week (the remaining contribution was from walking time with a mean of 1.8 h per week). This suggests a large proportion of this sample are not meeting current PA recommendations [56]. Along with low PA levels, Profile 1 also exhibited the highest sedentary time (6.9 h per day) and the lowest MIND diet adherence, further bolstering the rationale for intervention efforts to focus on such a subgroup.

Collectively, a sub-group characterised as “Moderate activity, non-smokers” (Profile 2) exhibited slight cognitive advantage over Profile 3 “Highly active, unhealthy drinkers” and Profile 1 “Inactive, poor diet”. Profile 2 was characterised by highest diet quality and high PA levels and hence this finding is consistent with findings from previous research reporting that inactivity and poor diet quality individually, are important modifiable factors in the prevention of cognitive decline and dementia [6, 57–60]. Profile 2 also exhibited a lower probability of being a current smoker which may also have contributed the observed cognitive benefits, since smoking is regarded as having one of the highest modifiable population attributable factors (PAF) for dementia [6]. Despite these findings, due to the cross-sectional nature of our study we cannot discount the possibility of reverse causality, whereby cognitive function impacts on engagement in lifestyle behaviours. Future research should aim to examine prospective associations between clustered lifestyle behaviours and subsequent incidence of cognitive decline and/or dementia.

One of the strengths of this study is the use of LPA to identify distinct person-centred sub-groups based on similar patterns of lifestyle behaviours. This presents a valuable method for identifying distinct sub-groups within a population that may be at increased risk of cognitive decline and dementia and providing insightful information around the clustering of key HRBs that can inform preventative strategies. However, some limitations of data-driven approaches such as LPA need to be acknowledged. While data-driven, there is still some subjectivity involved in the defining and operationalising of classes/profiles. Furthermore, the person-centred nature may have implications for generalisability of findings to wider population groups. A further limitation of this study that all lifestyle variables were based on self-report and hence may be subject to bias from under- or over-reporting. Furthermore, the AusDiab study was not designed with the intention of measuring dementia risk factors and did not include incident dementia as an outcome. As a result, there are other modifiable lifestyle behaviours, e.g., sleep, social engagement, and cognitive engagement that are not included in the current analyses and could further assist with characterising at-risk sub-groups. Despite these limitations the current study demonstrates an insightful example of how LPA can be applied in this field.

We believe that the use of advanced data-driven approaches to identify observed sub-groups based on similar lifestyle behaviours, and comparing these to key cognitive outcome measures complements previous work focused on the association between combined modifiable lifestyle factors and cognition [29]. While simple additive risk index approaches have great utility in informing individual-level intervention/risk reduction strategies, data-driven approaches such as LPA take into account possible synergistic, multiplicative or other relationships between modifiable factors, and may aid in identification of ‘at-risk’ populations to which to target public health risk reduction strategies for the prevention of cognitive decline and dementia. They may also be able to inform development and refinement of lifestyle-based non-pharmacological multidomain intervention strategies by providing specific insights around the behavioural clustering of population sub-groups, and particular behaviours that have largest scope for improvement.

## Conclusion

This study has demonstrated the ability of the data-driven approach LPA, to identify distinct subgroups of individuals based on key lifestyle behaviours and has identified that the “Moderate activity, non-smokers” group had better cognitive functioning compared to two other subgroups. As data-driven approaches such as LPA consider the complex interrelationships between lifestyle factors, their use in future research to elucidate subgroups at risk of cognitive decline/dementia and inform prioritisation of multidomain intervention strategies, is warranted.

## Electronic supplementary material

Below is the link to the electronic supplementary material.

Supplementary Material 1

## Data Availability

Data that support the findings of this study are available on request under a licence agreement. Written applications can be made to the AusDiab Steering Committee (Dianna.Magliano@baker.edu.au).

## List of abbreviations

AAS: Active Australia Survey
aBIC: adjusted Bayesian Information Criterion
AIC: Akaike Information Criterion
BCH: Bolck, Croon & Hagenaars approach
BIC: Bayesian Information Criterion
BLRT: Bootstrapped Likelihood Ratio Test
BMI: Body Mass Index
CCV: Cancer Council Victoria
CES-D: Center for Epidemiologic Studies Depression Scale
CVD: Cardiovascular Disease
CVLT: California Verbal Learning Test
DAG: Directed Acyclic Graph
DBP: Diastolic Blood Pressure
FFQ: Food Frequency Questionnaire
FP: Free Parameters
FPG: Fasting plasma glucose
LCA: Latent class analysis
LMR-LRT: Lo-Mendell-Rubin likelihood ratio test
LPA: Latent profile analysis
MCI: Mild Cognitive Impairment
MIND: Mediterranean-Dietary Approaches to Stop Hypertension Diet Intervention for Neurodegenerative Delay diet
NHMRC: National Health and Medical Research Council
PA: Physical Activity
PFA: PPopulation attributable factor
SBP: Systolic blood pressure
SDMT: Symbol-Digit Modalities Test
STW: Spot-the-word test
WHO: World Health Organization
