# Supplementary material for: Associations between data-driven lifestyle profiles and cognitive function in the AusDiab study
Source: BMC Public Health. 2022 Nov 29;22:1990. doi: 10.1186/s12889-022-14379-z (PMC9707000; doi:10.1186/s12889-022-14379-z)
Supplement: Supplementary file 1 — Supplementary Material 1 [file 12889_2022_14379_MOESM1_ESM.docx]

**Supplementary material**


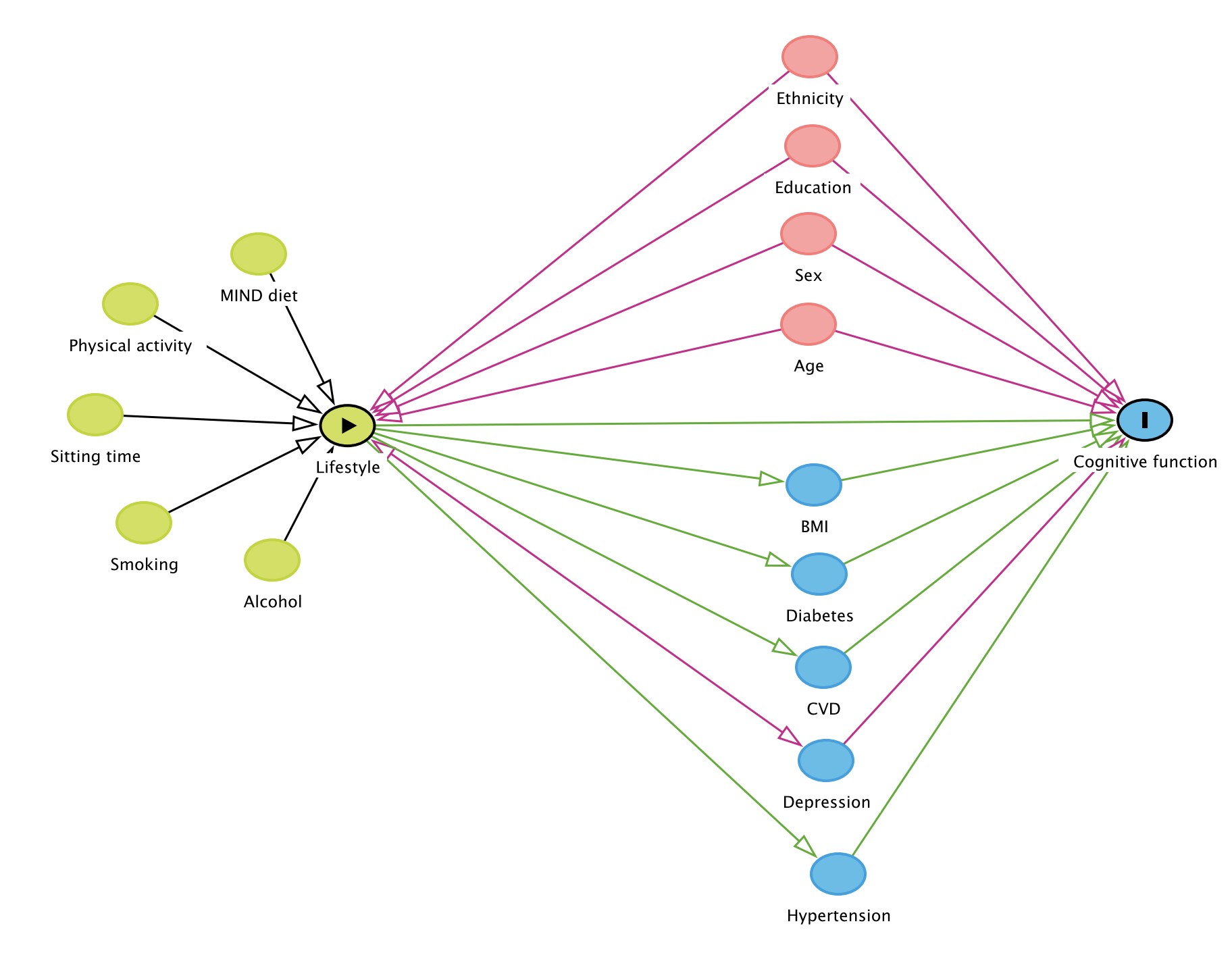


**Supplementary figure 1.** Directed Acyclic graph (DAG)

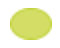
 ancestor of exposure;
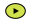
 exposure;
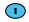
 outcome;
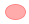
 ancestor of exposure and outcome;
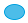
 ancestor of outcome;
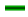
 casual path;
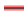
 biasing path
